# The rs1126616 Single Nucleotide Polymorphism of the Osteopontin Gene Is Independently Associated with Cardiovascular Events in a Chronic Kidney Disease Cohort

**DOI:** 10.3390/jcm8050592

**Published:** 2019-04-29

**Authors:** Serafí Cambray, Rajesh Kumar Galimudi, Milica Bozic, Marcelino Bermúdez-López, Isabel Rodríguez, José M. Valdivielso

**Affiliations:** 1 Vascular and Renal Translational Research Group, Biomedical Research Institute, IRBLLEIDA, and RedinRen-ISCIII, 25198 Lleida, Spain; seracc@yahoo.es (S.C.); rajeshgkumar26@gmail.com (R.K.G.); bozicm@medicina.udl.cat (M.B.); mbermudez@irblleida.cat (M.B.-L.); 2 Bone and Mineral Research Unit, RedinRen-ISCIII, Hospital Universitario Central de Asturias, Universidad de Oviedo, Instituto de Investigación Sanitaria del Principado de Asturias (ISPA), 33011 Oviedo, Spain

**Keywords:** atherosclerotic plaque, cardiovascular event, chronic kidney disease, Osteopontin, polymorphisms, Osteopontin gene (*SPP1*)

## Abstract

Chronic kidney disease (CKD) is associated with a higher risk of cardiovascular events (CVE), partly due to the higher burden of atherosclerosis. Circulating Osteopontin (OPN) levels have been also shown to have a potential role in the development of atherosclerosis. Indeed, CKD patients show an increase in circulating OPN levels, but their effect of CKD-related atherosclerosis is not clear. Polymorphisms in the OPN gene (*SPP1*) have been studied in atheromatous disease, but reported results show conflictive findings. Thus, the main aim of the present study is to analyze the influence of *SPP1* polymorphisms in CVE in CKD patients, taking into account circulating OPN levels. We followed 559 healthy controls and 2445 CKD patients without previous CVE from the National Observatory of Atherosclerosis in Nephrology study (NEFRONA study). After 48 months of follow-up 206 CVE were recorded. Genotyping for rs9138, rs1126616, rs1126772, rs11730582 and rs28357094 polymorphisms of the *SPP1* gene was performed along with the measurements of plasma OPN levels. The group of patients with CVE showed higher incidence of atherosclerotic plaque (90.3% vs 64.5%; *p* < 0.001) and higher OPN levels (*p* < 0.001) at baseline. Patients with the heterozygous genotype of the rs1126616 polymorphism showed a higher hazard ratio of having a CVE, even after adjustment for multiple potential confounders. After adjustment, OPN levels were no longer associated with the incidence of CVE. We found that the rs1126616 single nucleotide polymorphism (SNP) of the *SPP1* gene is independently associated with a higher incidence of CVE in a cohort of CKD patients and that it could be used to predict CVE risk.

## 1. Introduction

Chronic kidney disease (CKD) is associated with a higher risk for cardiovascular events (CVE) [1]. This increased risk seems to be strongly associated with electrolyte disturbances in dialysis patients, with a higher burden of atheromatous plaque playing a paramount role in the earlier stages [2,3]. In agreement with this hypothesis, atherosclerosis associated biomarkers showed good prognostic capacity to predict CVE in CKD patients [4], and the combination of some biomarkers associated with the presence of atheromatous plaque like Osteoprotegerin (OPG), Osteopontin (OPN) and soluble tumor necrosis factor-like weak inducer of apoptosis (sTWEAK) increased the accuracy of predictive models for cardiovascular outcomes in CKD patients [5].

OPN is an extracellular matrix glycoprotein produced by cells involved in bone morphogenesis, which plays a major role in the control of biomineralization and calcification. OPN is a secreted protein that contains several functional domains, including a number of integrin-interacting domains which facilitate the recruitment of inflammatory cells [6]. It is upregulated in atherosclerotic settings, where it is expressed by macrophages, endothelial and vascular smooth muscle cells, and modulates the inflammatory response by recruiting multiple inflammatory cell types [7,8,9]. Mice overexpressing OPN show smooth muscle cell hyperproliferation, medial thickening, and neointimal formation [10], and mice with OPN deficiency display attenuated atherosclerosis [11]. In clinical studies, increased plasma OPN expression levels are associated with the presence and severity of coronary artery disease [12,13] and with the levels of calcification [12,14]. Furthermore, the levels of OPN are also affecting the degree of neointima formation and of restenosis after revascularization, both in humans and in animal studies [10,15,16]. Finally, several studies have related high circulating levels of OPN to adverse cardiovascular events in the general population [17] and in CKD patients [18].

Apart from plasma OPN levels, single nucleotide polymorphisms (SNPs) on the *SPP1* gene (the gene encoding OPN), have also been associated with atheromatous disease and cardiovascular events [19]. For instance, rs28357094 located at the *SPP1* promoter has been shown to be related with increased carotid intima media thickness (cIMT) [20,21]. A second SNP, rs11730582 has also been associated with increased cIMT in stroke patients [21]. However, these results are questioned, as some researchers found no association between these polymorphisms and atherosclerotic markers [22,23]. Furthermore, most of the studies focusing in *SPP1* SNPs do not quantify plasma OPN, precluding further analysis on its effects on OPN levels.

In view of these data, we studied the association of SNPs in the *SPP1* gene and circulating levels of OPN with the incidence of CVE in a cohort of CKD patients during a 48 month period.

## 2. Experimental Section

### 2.1. Study Design and Participants

The NEFRONA (Observatory of Atherosclerosis in Nephrology) project is an observational, multi-center, prospective study that, from 2010 to 2012, recruited 2445 CKD patients from all over Spain [24,25]. Inclusion criteria were to present CKD stage 3 or higher (including patients in dialysis) and absence of any previous cardiovascular disease; CKD stage 3 was defined as glomerular filtration rate < 60 mL/min/1.73 m^2^ according to the 4-variable Modification of Diet in Renal Disease (MDRD) equation [26,27]. Exclusion criteria were life expectancy below 1 year, previous carotid surgery, acquired immune deficiency syndrome infection, having any organ transplant, any infection or hospital admission during the month previous to be included and pregnancy. We also included 559 controls (MDRD above 60 mL/min/1.73 m^2^) matched by age and sex randomly recruited from several Spanish Primary Care centers during the same period (2010 to 2012).

On the inclusion day, we obtained anthropometric, demographic, and biochemical data together with a comprehensive vascular ultrasound analysis to assess the presence of atherosclerotic plaque [28].

Volunteers underwent a minimum of 4 years of follow-up and CVE events (fatal and non-fatal) were recorded by the referring physician. CVE included unstable angina, myocardial infarction, transient ischemic attack, cerebrovascular accident, congestive heart failure, arrhythmia, peripheral artery disease, amputation due to vascular disease, aortic aneurysm, myocardial infarction, arrhythmia, congestive heart failure, stroke, mesenteric infarction, and sudden death. All patients were clearly informed, and signed an informed consent; the local Ethics Committee of each hospital approved the protocol.

### 2.2. SNP Genotyping

DNA was obtained from blood samples stored at the Biobank of the Spanish Renal Research Network (REDinREN) [29] using the QIAamp DNA Blood Kit and following manufacturer instructions. Genotyping was performed with matrix assisted laser desorption ionization time-of-flight mass spectrometry in the Sequenom MassARRAY platform^®^, in Centro de Genotipado-Plataforma de Recursos Biomoleculares y Bioinformáticos (CEGEN-PRB2) del Instituto de Salud Carlos III (Nodo de la Universidad de Santiago de Compostela, A Coruña, Spain). As a quality control, we eliminated SNP clusters or samples with low genotyping percentage and SNPs not meeting the Hardy–Weinberg Equilibrium (HWE). We included replicates of samples between plates and samples from the Coriell Institute Biorepository for genotyping quality assessment. For this study, we focused on five SNPs distributed along the *SPP1* gene in the 5′ upstream region (rs11730582 and rs28357094), the coding region (rs1126616) and the 3′ untranslated region (rs9138 and rs1126772).

### 2.3. Determination of OPN Levels

OPN serum levels were measured using MILLIPLEX^®^ MAP kits (EMD Millipore Corporation, Darmstadt, Germany) in a blinded manner. Minimum detectable levels were 37.7 pg/mL, intra-assay coefficient of variation 2%, and inter-assay coefficient of variation 12%.

### 2.4. Statistical Analysis

Qualitative variables were expressed as *n* (%), and quantitative variables as median (Q1;Q3) or mean (SD) depending on normality of distribution. Comparisons between groups were performed with Mann–Whitney U test or Student’s *t* test for non-normally or normally distributed quantitative variables, respectively, and Chi-squared test for categorical data.

To determine the genotypic association, genotype frequency, single SNP frequency under multiple inheritance models, haplotype frequency, and haplotype association with the response we used SNPStats [30]. Linkage disequilibrium (LD) was calculated with HAPLOVIEW [31] and compared to IBS population (Iberian Population in Spain) from Ensembl [32]. 

Kaplan–Meier curves were used to assess time to outcome according to a multiple markers score, and compared with the Mantel–Cox test. Time to event analysis was done using the Cox proportional hazards model, including adjustment for potential confounding factors. Final model was obtained through a forward stepwise method based on the likelihood ratio test. Data were presented as hazard ratios (HRs) and 95% confidence intervals (95% CIs), and *p* value < 0.05 was considered statistically significant.

## 3. Results

In the median follow-up period of 48.1 months, 206 CVE were reported (7.3%). The group of patients suffering a CVE were older, predominantly males, with a higher body mass index, systolic blood pressure and urine albumin to creatinine ratio, and showed an increased proportion of other classical cardiovascular risk factors (smoking, diabetes, hypertension, and dyslipemia). The group of patients suffering a CVE also displayed a higher percentage of patients in a more advanced CKD stage and with atheromatous plaque. Furthermore, they had lower levels of total, low-density lipoprotein (LDL) and high-density lipoprotein (HDL) cholesterol and higher triglycerides. In addition, blood levels of phosphorus, potassium, and ultra-sensitive c-reactive protein usCRP were higher whereas levels of 25(OH) vitamin D were lower. Levels of OPN were also significantly higher in the group of patients suffering a CVE (Table 1).

**Figure 1:**
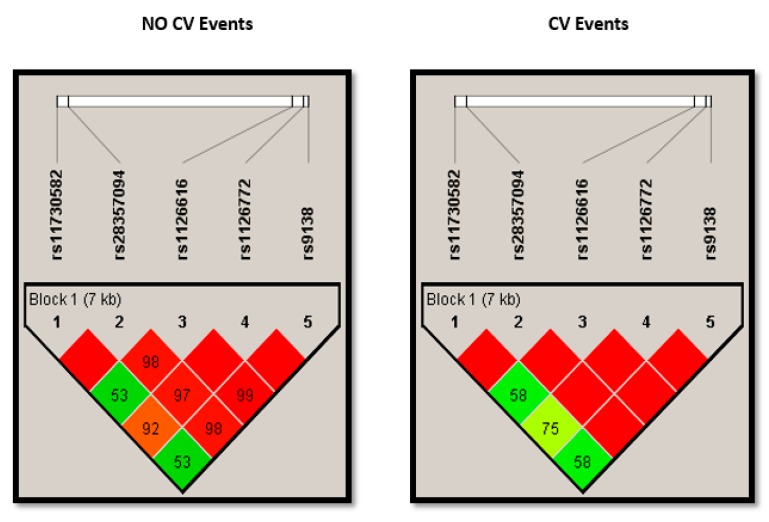
Plots showing linkage disequilibrium (LD) of alleles at different SNPs of *SPP1* in chronic kidney disease patients stratified according to the presence of cardiovascular events. SNPs: single nucleotide polymorphisms; *SPP1*: Osteopontin gene; CV: cardiovascular.

All the SNPs followed the HWE (Supplemental Table S1). We analyzed the crude association of the five SNPs (rs9138, rs1126616, rs1126772, rs11730582 and rs28357094) with CVE by testing five different models: co-dominant, dominant, recessive, over dominant and log-additive. Table 2 shows the three SNPs that were significantly associated with higher odds of having a CVE under the selected genetic model. Complete information for all SNPs and models is shown in Supplemental Tables S2–S6. For rs1126616 and rs9138, the over dominant models yielded significant differences, and for rs1126772 the dominant model returned the highest odds ratio and the lowest *p* value. As the three SNPs are in almost complete linkage disequilibrium (Figure 1), for further analysis we only considered the rs1126616, as the number of missing values was lower in this SNP.

**Figure 2:**
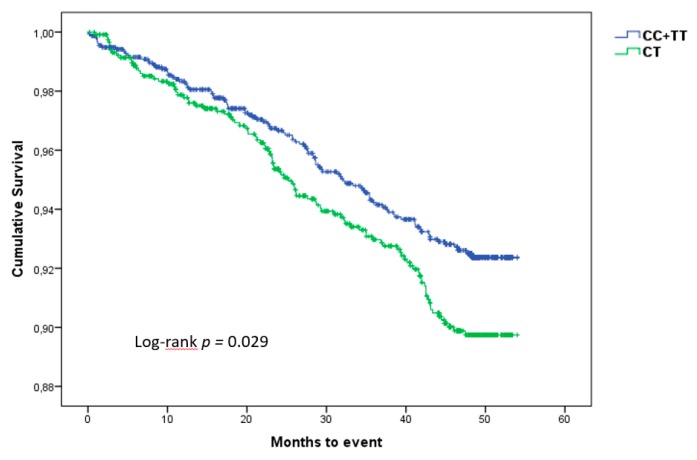
Kaplan–Meier curves showing the difference in the incidence of cardiovascular events according to genotype, during the follow-up period.

Taking into account the importance of rs1126616, we performed a Kaplan–Meier survival analysis to determine its effect on CVE. Figure 2 shows that patients with homozygote genotypes present a lower incidence of CVE than patients with the heterozygote genotype. The differences between both groups were assessed with a log-rank test, reaching a significant *p* value of 0.029.

**Figure 3:**
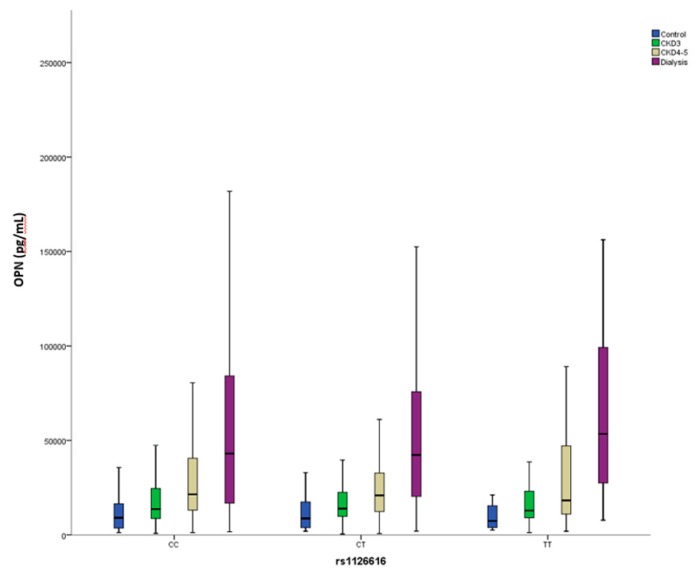
Circulating Osteopontin (OPN) levels stratified according to rs1126616 genotype and chronic kidney disease stage.

When our cohort was analyzed according to the proposed genetic model, significant differences were observed only for the incidence of CVE (Table 3). Notably, OPN levels were almost equal between both groups and were similarly modified by the CKD stage (Figure 3). 

Then we performed a multivariate proportional hazards Cox regression analysis including clinical and biochemical variables theoretically associated with a higher risk of presenting a CVE, together with the rs1126616 polymorphism. As shown in Table 4, variables with a statistically significant association with CVE were older age, the presence of diabetes and atheromatous plaque, higher levels of phosphate and c-reactive protein, and lower levels of HDL cholesterol and vitamin D. Heterozygosity for the rs1126772 genotype was associated with higher hazards of having a CVE.

Although the previous model was adjusted by CKD stage, we performed a sensitivity analysis eliminating the dialysis patients because they have a higher mortality rate. Thus, in Table 5 we show the Cox regression model further adjusted by proteinuria. The results show that the rs1126616 polymorphism is still significantly associated with the incidence of CVE.

## 4. Discussion

Cardiovascular events are one of the main complications of CKD patients, in which cardiovascular mortality is the main cause of death [33]. The causes of this excess of cardiovascular complications are not clear, although an increase in atherosclerotic-related problems has been suggested [3,34]. Accelerated atherosclerosis is a clinical feature associated with CKD [35], but the mechanisms involved are not fully understood. Among the metabolic alterations presented by CKD patients that could be involved in the excessive cardiovascular complications, changes in mineral metabolism are suggested to play a role [2,36,37]. OPN is a protein that is implicated in calcification, proliferation, and migration of vascular smooth muscle cells, providing a possible link between mineral metabolism and formation of atheroma plaque [38,39,40]. 

There is a clear association of OPN protein levels with atherosclerosis [6] and cardiovascular disease [41], and many studies have shown an association of higher OPN levels with CVE in several populations, including the NEFRONA cohort [5,42,43,44]. In our analysis, in agreement with previous reports, OPN levels increased progressively with the degree of renal function impairment [18], and were higher in patients with CVE. However, after adjustment for traditional risk factors and rs1126616, OPN levels were no longer significantly associated with the incidence of CVE. In any case, in the multivariate proportional hazards Cox regression analysis, the higher tertile of OPN almost reached statistical significance (Hazard Ratio: 2.9; *p* = 0.088; data not shown) so probably, the relatively low number of events precluded the analysis from having enough potency to show the association of OPN levels with CVE. 

Currently, there are few studies analyzing OPN polymorphisms and CVE. The results of the present study demonstrate that rs1126616 polymorphism of the *SPP1* gene is independently associated with a higher incidence of CVE in a cohort of CKD patients. A previous study from Brand-Herrmann and colleagues did not find any association of the *SPP1* rs1126616 SNP to myocardial or brain infarction [19], but differs from our study in many aspects. The NEFRONA study is a prospective cohort study of mainly CKD patients, and our endpoint (CVE) is a composite containing all cardiovascular events. Norman et al. investigated the association between OPN SNPs and abdominal aortic aneurysm (AAA), and found no significant associations between rs1126616 and AAA or aortic diameter, neither with aortic expansion nor with OPN levels [45]. Again, the fact that their cohort was not composed of CKD patients, and that they only focus on AAA could explain differences between both studies. It is worth noting that none of these studies [19,45] tested different genetic models of inheritance, precluding the identification of the over dominant genotype that arose in our study. Usually genetic disease models follow dominant, recessive, or co-dominant models, but over dominant models have been also described in many studies [46,47,48,49]. 

Apart from CVE, the rs1126616 polymorphism has also been found to be associated with CKD [50], urolithiasis [51], and to the prevalence of lupus nephritis [52]. However, no study has previously shown the effect of the SNP on circulating levels of OPN in patients with different stages of CKD. Previous reports have shown no differences in OPN levels between different genotypes of the SNP [51,52,53]. Our results agree with the previous literature as the different genotypes do not modify the OPN levels, and more advanced stages of CKD show increased levels of OPN. The rs1126616 is not expected to change either the expression of the gene or the sequence of the protein as the SNP is a synonymous change (p.Ala236=) located at the coding region. In our population, rs1126616 is in almost complete LD with the other two SNPs that, consequently, yielded statistically significant differences in the univariate analysis (rs1126772 and rs9138). These two SNPs are located at the 3′ untranslated region but in our study, and in agreement with previous reports [51,52,53], the OPN levels did not change according to these genotypes. Therefore, we cannot rule out that the association found between this SNP and the presence of CVE could be due to another functional SNP in LD with the one we studied.

One limitation of our work is the lack of a validation cohort. As a second limitation, previous Genome-Wide Association Studies (GWAS) did not find any association between any of the present polymorphisms and cardiovascular disease [54]. However, this could be due to the very strict limitations applied to *p* values in those kind of studies. As for strengths of the current work, this is the first time that *SPP1* gene polymorphisms have been analyzed in a relatively large CKD population on which OPN levels have been related to phenotype and genotype. Moreover, the many clinical and biochemical data of NEFRONA study allowed us to better adjust and to put into context the effect of the selected SNPs. 

In conclusion, the rs1126616 SNP of the *SPP1* gene is independently associated with cardiovascular events in a cohort of CKD patients regardless of OPN levels and thus, if validated, could be used to predict CVE risk in CKD patients.

## Figures and Tables

**Table 1:** Epidemiological, clinical, and biochemical parameters of patients according to the presence or absence of cardiovascular events.

Variable			No CVE	CVE	*p* Value
			*n* = 2609	*n* = 206	
Age (years)			59 (48;67)	64.5 (58;70)	<0.001
Sex (Female)			1056 (40.4)	60 (29)	0.001
Body mass index (kg/m^2^)			27.6 (24.6;31)	28.9 (25.7;32.12)	0.005
Smoking (Yes)			1481 (56.7)	137 (66.5)	0.006
Diabetes (Yes)			546 (20.9)	88 (42.7)	<0.001
Hypertension (Yes)			2068 (79.2)	191 (92.7)	<0.001
Dyslipemia (Yes)			1551 (59.4)	149 (72.3)	<0.001
Systolic blood pressure (mmHg)			140 (21.3)	148 (24.7)	<0.001
Dyastolic blood pressure (mmHg)			81 (11)	81 (13)	0.556
Chronic kidney disease Stage	Control		536 (20.5)	13 (6.3)	<0.001
	Stage 3		819 (31.4)	66 (32)	
	Stage 4–5		684 (26.2)	62 (30.1)	
	Dialysis		570 (21.8)	65 (31.6)	
Presence of plaque at baseline			1684 (64.5)	186 (90.3)	<0.001
Total Cholesterol (mg/dL)			182 (156.5;208)	171 (141.2;207)	0.017
HDL Cholesterol (mg/dL)			48 (39;59]	44 (35;52)	<0.001
LDL Cholesterol (mg/dL)			105 (83;128)	98 (72;128)	0.042
Triglycerides (mg/dL)			118 (86.2;166)	138 (100;181)	<0.001
Glucose (mg/dL)			96 (87;110)	105 (91.6;139.2)	<0.001
Calcium (mg/dL)			9.3 (8.8;9.5)	9.3 (8.9;9.7)	0.095
Phosphate (mg/dL)			3.8 (3.3;4.4)	4 (3.4;5)	0.003
Sodium (mEq/L)			140.6 (139;142)	140 (138;142)	0.015
Potassium (mEq/L)			4.7 (4.37;5.1)	4.9 (4.4;5.3)	<0.001
C-reactive protein (mg/L)			4.2 (8.2)	6.2 (12)	0.001
ACR (mgAlb/gCreat)		ACR < 30	398 (34.3)	20 (22.2)	0.02
		ACR = 30–300	416 (35.9)	32 (35.6)	
		ACR > 300	346 (29.8)	38 (42.2)	
25(OH) vitamin D (ng/mL)			17.1 (7.8)	14.6 (6.9)	<0.001
OPN (ng/mL)			16.7 (9.3;33)	22.6 (11.9;53.3)	<0.001

Qualitative variables are expressed as *n* (%) and quantitative variables as median (Q1;Q3) or mean (SD) depending on the normality of the distribution. Comparisons between groups performed with Mann–Whitney U test or Student’s *t* test for quantitative non-normally distributed variables and normally distributed respectively, and Chi-squared test for categorical data. CVE, cardiovascular events; HDL, high-density lipoprotein; LDL, low-density lipoprotein; ACR, urine albumin to creatinine ratio; OPN, Osteopontin.

**Table 2:** Selected models of inheritance for the three *SPP1* polymorphisms associated with the presence of cardiovascular events.

SNP (*n*)	Model	Genotype	NO CVE *n* (%)	CVE *n* (%)	Odds Ratio (95% CI)	*p* Value
rs1126616(2815)	Over dominant	CC-TT	1512 (58)	102 (49.5)	1.00	0.019
		CT	1097 (42)	104 (50.5)	1.41 (1.06–1.87) *	
rs1126772(1613)	Dominant	AA	903 (59.9)	49 (46.2)	1.00	0.006
		AG-GG	604 (40.1)	57 (53.8)	1.74 (1.17–2.58) *	
rs9138(2815)	Over dominant	AA-CC	1549 (59.3)	106 (51.5)	1.00	0.028
		AC	1062 (40.7)	100 (48.5)	1.38 (1.04–1.83) *	

* Significant odds ratio. Odds ratio is referred to dominant homozygote for each model. The *p* value is for model performance for each individual polymorphism against other possible models (co-dominant, dominant, recessive, over dominant, log-additive). CVE, cardiovascular event; SNP: single nucleotide polymorphisms; 95% CIs: 95% confidence intervals; *SPP1*: Osteopontin gene.

**Table 3:** Association of *SPP1* rs1126616 C > T polymorphism with epidemiological, clinical and biochemical parameters of the cohort.

Variables			GENOTYPES	GENOTYPE	*p* Value
			CC + TT	CT	
			*n* = 1555	*n* = 1168	
Age (years)			56.36(12.48)	57.96(12.48)	0.219
Sex (Female)			619(39.8)	457(39.1)	0.719
Body mass index (kg/m^2^)			28.12(5)	28.38(5.1)	0.186
Smoking (Yes)			886(57)	689(59)	0.293
Diabetes (Yes)			357(23)	253(21)	0.442
Hypertension (Yes)			1225(78.8)	953(81.6)	0.069
Dyslipemia			946(60.8)	703(60.2)	0.732
Systolic blood pressure (mmHg)			140.99(21.47)	141.17(21.71)	0.823
Dyastolic blood pressure (mmHg)			81.14(11.39)	80.93(11.34)	0.627
Chronic kidney disease Stage	Control		334(21.5)	205(17.6)	0.058
	Stage 3		485(31.2)	382(32.7)	
	Stage 4–5		413(26.6)	310(26.5)	
	Dialysis		323(20.8)	271(23.2)	
Cardiovascular Events			100(6.4)	101(8.6)	0.029
Any basal plaque (yes)			1048(67.4)	785(67.2)	0.918
Total Cholesterol (mg/dL)			183.71(40,44)	182.66(38.73)	0.5
HDL Cholesterol (mg/dL)			50.45(15.49)	50.12(15.41)	0.606
LDL Cholesterol (mg/dL)			107.32(35.21)	106.42(34.08)	0.534
Triglycerides (mg/dL)			136.58(76.54)	140.85(81.53)	0.17
C-reactive protein			4.33(8.55)	4.34(8.26)	0.964
ACR (mgAlb/gCreat)		ACR < 30	239 (34.9)	168 (31.2)	0.390
		ACR = 30–300	242 (35.3)	201 (37.3)	
		ACR > 300	204 (29.8)	170 (31.5)	
Glucose (mg/dL)			106.76(39.22)	105.91(34.23)	0.55
Calcium (mg/dL)			9.32(0.58)	9.35(0.55)	0.18
Phosphate (mg/dL)			3.97(1.06)	3.96(1.05)	0.768
Sodium (mEq/L)			140.36(2.97)	140.51(2.98)	0.176
Potassium (mEq/L)			4.75(0.6)	4.74(0.56)	0.642

*p* value < 0.05 boldface typed. Qualitative variables are expressed as n (%) and quantitative variables as mean (SD). Comparisons between groups performed with Student’s *t* test for quantitative variables, and Chi-squared test for categorical data. HDL, high-density lipoprotein; LDL, low-density lipoprotein; ACR, urine albumin to creatinine ratio; OPN, Osteopontin.

**Table 4:** Cox regression modelling the incidence of CVE including dialysis patients (*n* = 2093).

Variables	HR (95% CI)	*p* Value
Age (years)	1.020 (1.003–1.038)	0.024
Diabetes	1.705(1.23–2.36)	0.001
HDL Cholesterol (mg/dL)	0.983(0.972–0.995)	0.005
Phosphate (mg/dL)	1.387(1.218–1.579)	<0.001
25(OH) vitamin D (ng/mL)	0.95(0.924–0.976)	<0.001
C-reactive protein	1.013(1.001–1.024)	0.037
Plaque presence	2.555(1.461–4.468)	0.001
rs1126616	1.462(1.067–2.004)	0.018

Variables that did not reach significance level: Sex, BMI, Smoker, Hypertension, Dyslipemia, Systolic blood pressure (SBP), Diastolic blood pressure (DBP), CKD stage, Total cholesterol, LDL cholesterol, triglycerides, glucose, calcium, and OPN tertile. HR, Hazard Ratio. CI: Confidence Intervals.

**Table 5:** Cox regression modelling the incidence of CVE excluding dialysis patients (*n* = 1021).

Variables	HR (95% CI)	*p* Value
Diabetes	1.913(1.196–3.060)	0.007
HDL Cholesterol (mg/dL)	0.975(0.957–0.994)	0.008
Plaque presence	2.438(1.265–4.697)	0.008
C-reactive protein	1.019(1.005–1.034)	0.01
rs1126616	1.626(1.026–2.576)	0.039

Variables that did not reach significance level: Age, Sex, BMI, Smoker, Hypertension, Dyslipemia, SBP, DBP, CKD stage, Total cholesterol, LDL cholesterol, triglycerides, albumin to creatinine ratio, glucose, calcium, phosphate, 25(OH) vitamin D and OPN tertile. HR: Hazard Ratio; CI: Confidence Intervals.

## Supplementary Materials

The following are available online at https://www.mdpi.com/2077-0383/8/5/592/s1, Table S1: HWE equilibrium for the five *SPP1* SNPs analyzed. Table S2: Genetic polymorphism of *SPP1* rs1126772 A/G in association with CVE in CKD patients. Table S3: Genetic polymorphism of *SPP1* rs1126616 C/T in association with CVE in CKD patients. Table S4: Genetic polymorphism of *SPP1* rs9138 A/C in association with CVE in CKD patients. Table S5: Genetic polymorphism of *SPP1* rs11730582 C/T in association with CVE in CKD patients. Table S6: Genetic polymorphism of *SPP1* rs28357094 G/T in association with CVE in CKD patients.
