# Supplementary material for: The rs1126616 Single Nucleotide Polymorphism of the Osteopontin Gene Is Independently Associated with Cardiovascular Events in a Chronic Kidney Disease Cohort
Source: J Clin Med. 2019 Apr 29;8(5):592. doi: 10.3390/jcm8050592 (PMC6571716; doi:10.3390/jcm8050592)
Supplement: Supplementary file 1 [file jcm-08-00592-s001.pdf]

SUPPLEMENTAL FILES

**Supplemental Table S1:** Hardy–Weinberg equilibrium for the five *SPP1* SNPs analyzed.							
	**Homozygote Reference**		**Heterozygote**		**Homozygote variant**		***p* value**
	**Observed**	**Expected**	**Observed**	**Expected**	**Observed**	**Expected**	
**rs9138**	1352	1348.6	1130	1136.8	243	239.6	0.753
**rs1126616**	1312	1320.2	1168	1151.7	243	251.2	0.459
**rs11730582**	223	236.3	535	508.3	260	273.3	0.094
**rs28357094**	133	128.8	798	806.5	1267	1262.8	0.622
**rs1126772**	908	915.9	570	554.3	76	83.9	0.263
Comparison between groups by means of a Chi-squared test.							

Supplemental Table S2: Genetic polymorphism of *SPP1* rs1126772 A>G in association with CVE in chronic kidney disease (CKD) patients.

**Model**	**Genotype**	**No CVE N = 1507 *n* (%)**	**CVE N = 106 *n* (%)**	**Odds ratio (95% CI)**	***p* value**	**AIC**
**Co-dominant**	AA	903 (59.9%)	49 (46.2%)	1.00	0.016	779.8
	AG	529 (35.1%)	52 (49.1%)	1.81 (1.21–2.72)*		
	GG	75 (5%)	5 (4.7%)	1.23 (0.48–3.18)		
**Dominant**	AA	903(59.9)	49(46.2)	1.00	0.006	778.5
	AG-GG	604(40.1)	57(53.8)	1.74 (1.17–2.58)*		
**Recessive**	AA-AG	1432(95)	101(95.3)	1.00	0.9	786
	GG	75(5)	5(4.7)	0.95 (0.37–2.39)		
**Over dominant**	AA-GG	978(64.9)	54(50.9)	1.00	0.004	778
	AG	529(35.1)	52(49.1)	1.78 (1.20–2.64)*		
**Log-Additive**	**---**	---	---	1.43 (1.05–1.96)*	0.027	781.1
**HWE (*p* value)**	**---**	0.88	0.098	---	---	---

*Significant odds ratio. The *p* value is obtained by comparison of Akaike information criterion (AIC) from different models; CVE, cardiovascular events; HWE, Hardy–Weinberg equilibrium.

Supplemental Table S3: Genetic polymorphism of *SPP1* rs1126616 C>T in association with CVE in CKD patients.

**Model**	**Genotype**	**No CVE N = 2609 *n* (%)**	**CVE N = 206 *n* (%)**	**Odds ratio (95% CI)**	***p* value**	**AIC**
**Co-dominant**	CC	1276 (48.9%)	87 (42.2%)	1.00	0.062	1474.3
	CT	1097 (42%)	104 (50.5%)	1.39 (1.03–1.87)*		
	TT	236 (9.1%)	15 (7.3%)	0.93 (0.53–1.64)		
**Dominant**	CC	1276 (48.9%)	87 (42.2%)	1.00	0.064	1474.4
	CT-TT	1333 (51.1%)	119 (57.8%)	1.31 (0.98–1.74)		
**Recessive**	CC-CT	2373 (91%)	191 (92.7%)	1.00	0.38	1477.1
	TT	236 (9.1%)	15 (7.3%)	0.79 (0.46–1.36)		
**Over dominant**	CC-TT	1512 (58%)	102 (49.5%)	1.00	0.019	1472.4
	CT	1097 (42%)	104 (50.5%)	1.41 (1.06–1.87)*		
**Log-Additive**	---	---	---	1.12 (0.90–1.39)	0.3	1476.8
**HWE (*p* value)**	---	1	0.039	---	---	---

*Significant odds ratio. The *p* value is obtained by comparison of Akaike information criterion (AIC) from different models; CVE, cardiovascular events.

**Supplemental Table S4:** Genetic polymorphism of *SPP1* rs9138 A>C in association with CVE in CKD patients.						
**Model**	**Genotype**	**No CVE N = 2609 *n* (%)**	**CVE N = 206 *n* (%)**	**Odds ratio (95% CI)**	***p* value**	**AIC**
**Co-dominant**	AA	1313 (50.3%)	91 (44.2%)	1.00	0.086	1475.3
	AC	1062 (40.7%)	100 (48.5%)	1.36 (1.01–1.83)*		
	CC	236 (9%)	15 (7.3%)	0.92 (0.52-1.61)		
**Dominant**	AA	1313 (50.3%)	91 (44.2%)	1.00	0.091	1475.3
	AC-CC	1298 (49.7%)	115 (55.8%)	1.28 (0.96–1.70)		
**Recessive**	AA-AC	2375 (91%)	191 (92.7%)	1.00	0.38	1477.4
	CC	236 (9%)	15 (7.3%)	0.79 (0.46–1.36)		
**Over dominant**	AA-CC	1549 (59.3%)	106 (51.5%)	1.00	0.028	1473.3
	AC	1062 (40.7%)	100 (48.5%)	1.38 (1.04–1.83)*		
**Log-Additive**	---	---	---	1.11 (0.89–1.37)	0.36	1477.3
**HWE (*p* value)**	---	0.32	0.11	---	---	---
*Significant odds ratio. The *p* value is obtained by comparison of Akaike information criterion (AIC) from different models. CVE, cardiovascular events.						

**Supplemental Table S5:** Genetic polymorphism of *SPP1* rs11730582 C>T in association with CVE in CKD patients.						
**Model**	**Genotype**	**No CVE N = 2609 *n* (%)**	**CVE N = 206 *n* (%)**	**Odds ratio (95% CI)**	***p* value**	**AIC**
**Co-dominant**	CC	224(22.4%)	12(23.1%)	1.00	0.72	419.3
	CT	514(51.5%)	29(55.8%)	1.05 (0.53–2.10)		
	TT	260(26.1%)	11(21.1%)	0.79 (0.34–1.82)		
**Dominant**	CC	224(22.4%)	12(23.1%)	1.00	0.92	417.9
	CT-TT	774(77.6%)	40(76.9%)	0.96 (0.50–1.87)		
**Recessive**	CC-CT	738(74%)	41(78.8%)	1.00	0.42	417.3
	TT	260(26.1%)	11(21.1%)	0.76 (0.39–1.50)		
**Over dominant**	CC-TT	484(48.5%)	23(44.2%)	1.19 (0.68–2.08)	0.55	417.6
	CT	514(51.5%)	29(55.8%)			
**Log-Additive**	---	---	---	0.89 (0.60–1.33)	0.58	417.6
**HWE (*p* value)**	---	0.34	0.58	---	---	
*Significant odds ratio. The *p* value is obtained by comparison of Akaike information criterion (AIC) from different models.						

**Supplemental Table S6:** Genetic polymorphism of *SPP1* rs28357094 G/T in association with CVE in CKD patients						
**Model**	**Genotype**	**No CVE N = 2609 *n* (%)**	**CVE N = 206 *n* (%)**	**Odds ratio (95% CI)**	***p* value**	**AIC**
**Co-dominant**	GG	126(6%)	8(5.3%)	1.00	0.7	1113.2
	GT	751(35.7%)	59(39.1%)	1.24 (0.58–2.65)		
	TT	1225(58.3%)	84(55.6%)	1.08 (0.51–2.28		
**Dominant**	GG	126(6%)	8(5.3%)	1.00	0.72	1111.7
	GT-TT	1976(94%)	143(94.7%)	1.14 (0.55–2.38)		
**Recessive**	GG-GT	877(41.7%)	67(44.4%)	1.00	0.52	1111.5
	TT	1225(58.3%)	84(55.6%)	0.90 (0.64–1.25)		
**Over dominant**	GG-TT	1351(64.3%)	92(60.9%)	1.00	0.41	1111.2
	GT	751(35.7%)	59(39.1%)	1.15 (0.82–1.62)		
**Log-Additive**	---	---	---	0.95 (0.73–1.24)	0.7	1111.7
**HWE (*p* value)**	---	0.44	0.67	---	---	
*Significant odds ratio. The *p* value is obtained by comparison of Akaike information criterion (AIC) from different models.						
